# Condições pós-COVID-19 no Sistema Único de Saúde: explorando o terreno incerto da identificação e enfrentamento

**DOI:** 10.1590/0102-311XPT046524

**Published:** 2024-08-23

**Authors:** Pollyanna Kassia de Oliveira Borges, Eliseu Alves Waldman, Camila Marinelli Martins

**Affiliations:** 1 Universidade Estadual de Ponta Grossa, Ponta Grossa, Brasil.; 2 Universidade de São Paulo, São Paulo, Brasil.; 3 AAC&T Research Consulting LTDA, Curitiba, Brasil.

Caras Editoras,

*Cadernos de Saúde Pública* (CSP) apresentou, no Editorial de fevereiro de 2024 ^1^, desafios para o Sistema Único de Saúde (SUS) referentes à COVID longa, tema de inegável atualidade mas insuficientemente explorado em nosso país. Os comentários se fundamentam em dois estudos ^2^ ^,^ ^3^ com foco na prevalência de sintomas pós-COVID-19, divulgados no mesmo número do periódico.

Esta carta objetiva comentar como os resultados publicados ^2^ ^,^ ^3^ contribuem no conhecimento do impacto para o SUS e no esclarecimento de dúvidas e controvérsias sobre a COVID longa, ou condições pós-COVID-19 ^4^. Para aprofundar a discussão, também apresentamos a prevalência das condições pós-COVID-19 em estudo realizado no Sul do Brasil, comparando diferentes critérios de definição de caso e confirmando as dificuldades acerca da variabilidade das medidas de condições pós-COVID-19 e a necessidade de pesquisas populacionais ^1^.

O estudo conduzido no Ceará, Brasil ^2^, mostrou que fadiga, alteração de memória e dispneia foram os principais sintomas de condições pós-COVID-19 sentidos por, respectivamente, 46%, 39% e 31% dos participantes. Os resultados expõem como as condições pós-COVID-19 repercutem no cotidiano das pessoas e abre discussão para os seus impactos laborais e econômicos. A ausência de retorno ao trabalho por causa das condições pós-COVID-19 se associou a piores indicadores e produziram custo indireto elevado, com mais de um terço das pessoas não conseguindo retornar ao trabalho 15 meses após a COVID-19. Essas demandas podem estar invisíveis à Previdência e à sociedade, e com atendimentos pouco planejados nas redes assistenciais do SUS.

No estudo do Mato Grosso, Brasil ^3^, a prevalência foi de 60% para 1-2 sintomas contínuos no primeiro ano pós-COVID-19. Mulheres, pessoas de 50-59 anos e os mais pobres tiveram maior proporção de pessoas com 3 ou mais sintomas de condições pós-COVID-19 após um ano. Os achados confirmam a desigualdade de gênero, idade e renda. As condições pós-COVID-19 se associaram a doenças crônicas preexistentes, mostrando que ampliam a carga de morbidade e devem ser incluídas no espectro da política pública de enfrentamento das doenças crônicas.

Ambos os estudos ^2^ ^,^ ^3^ revelam prevalência de condições pós-COVID-19 superiores aos 10%-20% estimados pela Organização Mundial da Saúde (OMS) ^5^, mas foram realizados com pacientes hospitalares ou em reabilitação. Persistem dúvidas se os achados de frequência e distribuição seriam equivalentes na população em geral brasileira, com casos leves e moderados da fase aguda da COVID-19 e alta cobertura vacinal. Os dados foram coletados em 2021 e no início de 2022, e não pudemos visualizar o impacto sobre as condições pós-COVID-19 após a entrada da variante ômicron no país. O Brasil deve estudar profundamente o acesso aos serviços de saúde pelas pessoas com condições pós-COVID-19, anos de vida perdidos, além da duração e sobreposição com outras condições de saúde e mortalidade (direta e indireta). É importante alcançarmos o entendimento mínimo da magnitude do problema no país para que o SUS elabore estratégias de vigilância e enfrentamento e esteja apto a elencar prioridades segundo futuras classificações de risco para as condições pós-COVID-19, que parecem ainda não ter se tornado realidade concreta para a sociedade e os governos a despeito do sofrimento individual dos doentes.

O registro de manifestações tardias de doenças infecciosas já foi descrito para outras doenças infecciosas, a síndrome pós-pólio, por exemplo. Porém, as condições pós-COVID-19 apresentam maior impacto nos sistemas de saúde em virtude do elevado número de casos de COVID-19 ocorridos em todo globo em um curto período. Consequentemente, as condições crônicas após a fase aguda da doença, ou mesmo da infecção oligo ou assintomática pelo SARS-CoV-2, podem ser esperadas em elevada proporção, com manifestações graves e limitadoras como mencionado nos estudos comentados ^2^ ^,^ ^3^.

Sem dúvida, o Editorial ^1^ acerta ao apontar a COVID longa como “*agenda inacabada do SUS*”. Os desafios a serem enfrentados são enormes, e as dificuldades passam pela padronização da nomenclatura, definição de caso, ausência de dados nacionais, sobreposição de sintomas, variabilidade na apresentação clínica dos casos, desenvolvimento de tratamentos, e outros. A seguir, comentamos sobre o desafio para definir os casos de condições pós-COVID-19. Variadas definições podem ter maior ou menor sensibilidade, além de dificultar o autorreconhecimento e o diagnóstico nos serviços SUS.

Os dois artigos publicados ^2^ ^,^ ^3^ empregaram a definição do Instituto Nacional de Excelência em Saúde e Cuidados do Reino Unido (NICE, acrônimo em inglês) ^6^, que classifica as populações em: COVID-19 aguda (0-4 semanas pós-COVID-19); sintomáticos contínuos (4-12 semanas); e síndrome pós-COVID-19 (≥ 12 semanas). O Ministério da Saúde ^4^ padronizou a definição de caso de acordo com o Centro de Controle e Prevenção de Doenças dos Estados Unidos (CDC, acrônimo em inglês), e diz que são “(...) *condições que continuam ou se desenvolvem quatro semanas ou mais após a infecção inicial* (...)”. A OMS ^5^ apresenta temporalidade distinta da anterior, e há ainda tentativas de classificação clínica a partir de score atribuído aos sintomas mais comuns ^7^.

Diferentes definições de caso dificultam estimativas da magnitude do problema. Além das muitas classificações existentes, há desafios internos das classificações. O NICE ^6^ indica que o termo “COVID longa” se refere tanto às subpopulações dos “sintomáticos contínuos” quanto da “síndrome pós-COVID-19”. Alguns autores assim procedem, e outros descrevem a frequência no critério NICE com base naqueles que apresentam a temporalidade da síndrome pós-COVID-19.

A OMS ^5^ (p. 1) determina que pessoas com condições pós-COVID-19 têm sintomas “*geralmente 3 meses após o início da COVID-19, que duram pelo menos 2 meses, e não podem ser explicados por um diagnóstico alternativo*”. Essa definição indica incerteza temporal, mencionando a duração de dois meses sem especificar se aqueles que iniciaram no *t0* com sintomas e perduraram sintomáticos dois meses, mas não completaram três meses após a infecção aguda, seriam casos. Ou até se os casos seriam as pessoas que iniciaram no *t0* e permaneceram doentes até o terceiro mês, independentemente da duração de uma semana, um mês ou mais após o terceiro mês, ou simplesmente se deve contar os doentes a partir de três meses pós-COVID-19. Todas as definições citadas são consideradas temporárias, mas pesquisadores as têm empregado nas pesquisas e nem sempre esclarecem se estão considerando o início dos sintomas, a duração ou ambos.

Em estudo, ainda em desenvolvimento (CAAE: 52790021.4.0000.0105), comparamos diferentes definições de caso com os dados de 1.721 pessoas que tiveram COVID-19 confirmada por RT-PCR no Sul do Brasil. Para a OMS ^5^, 78% dos entrevistados eram casos de condição pós-COVID-19. Segundo o Ministério da Saúde ^4^, eram 81,7%. No NICE ^6^, seriam 81,3% se considerando sintomáticos contínuos e síndrome pós-COVID-19, ou 77% se inserindo somente a síndrome pós-COVID-19 como casos. Menos de 40% atingiram o ponto do escore *Postacute Sequelae of SARS-CoV-2 Infection* (PASC; Sequelas Pós-agudas da Infecção por SARS-CoV-2) ^7^ (Tabela 1).

**Tabela 1:** Frequências de participantes segundo as definições de condições pós-COVID-19 - amostra balanceada para proporções da base populacional. *COVID Longa Ponta Grossa*, Ponta Grossa, Paraná, Brasil, 2023.

Variável	n	%	IC95%
COVID longa (de acordo com a OMS ^5^)			
Não	356	21,58	14,83-28,32
Sim	1.292	78,42	71,68-85,17
COVID longa (de acordo com o Ministério da Saúde ^4^)			
Não	308	18,71	12,38-25,04
Sim	1.340	81,69	74,96-87,62
PASC ^7^			
Não	1.150	66,84	58,90-74,77
Sim	570	33,16	25,23-41,10
NICE ^6^			
Não	236	14,31	8,70-19,92
COVID aguda	73	4,40	3,45-5,34
Sintomático contínuo	116	7,06	5,85-8,27
Síndrome pós-COVID-19	1.223	74,23	66,89-81,58

IC95%: intervalo de 95% de confiança; NICE: Instituto Nacional de Excelência em Saúde e Cuidados do Reino Unido; OMS: Orgnaização Mundial da Saúde; PASC: *Postacute Sequelae of SARS-CoV-2 Infection* (Sequelas Pós-agudas da Infecção por SARS-CoV-2).

Constatamos pequenas diferenças de prevalência entre as classificações, exceto para o PASC. Confirmamos a elevada proporção de casos a despeito do critério empregado, e nossos achados seguem na mesma direção dos estudos apresentados em CSP ^2^ ^,^ ^3^. A diferença de proporção entre os critérios OMS ^5^ e Ministério da Saúde ^4^ foi de 4%. Sem analisar as limitações de validade externa do nosso estudo, se considerarmos que 38 milhões de pessoas tiveram COVID-19 no Brasil, teríamos mais ou menos 1,5 milhões de brasileiros doentes para condições pós-COVID-19 de acordo com o critério empregado. Se um clínico utilizasse o escore PASC ^7^, por exemplo, deixaria de diagnosticar a metade dos doentes pelos critérios OMS ^5^ e Ministério da Saúde ^4^.

Conhecimento aprofundado sobre as condições pós-COVID-19 e solidez nas definições de caso trarão confiança e comparabilidade aos dados epidemiológicos. Os sistemas de vigilância poderão indicar as prioridades para outros setores da saúde com robustez. Incentivamos que novas publicações em CSP, e demais periódicos de Saúde Coletiva, continuem explorando a descrição e associações das condições pós-COVID-19. Mesmo que a prevalência real não seja conhecida, as condições pós-COVID-19 exigem imediato diagnóstico e tratamento nos serviços do SUS.
